# *SLCO1B1* c.388A > G variant incidence and the severity of hyperbilirubinemia in Indonesian neonates

**DOI:** 10.1186/s12887-019-1589-1

**Published:** 2019-06-28

**Authors:** Radhian Amandito, Rinawati Rohsiswatmo, Michelle Halim, Vanessa Tirtatjahja, Amarila Malik

**Affiliations:** 1 Neonatal Intensive Care Unit, Pondok Indah General Hospital, Jl Metro Duta Kav. UE, Pondok Indah, Jakarta, Indonesia; 2 Division of Perinatology, Department of Child Health, Cipto Mangunkusumo General Hospital, Faculty of Medicine Universitas Indonesia, Jl Pangeran Diponegoro No. 71, Central Jakarta, Jakarta, Indonesia; 3 Division of Pharmaceutical Microbiology and Biotechnology, Faculty of Pharmacy, Universitas Indonesia, UI Depok Campus, Depok, West Java 16436 Indonesia

**Keywords:** Bilirubin, Indonesia, neonatal hyperbilirubinemia, PCR-RFLP, Polymorphism, SLCO1B1

## Abstract

**Objective:**

It has been established that genetic factors play a substantial role in the development of neonatal hyperbilirubinemia. The population of Indonesia and other Southeast Asian countries has similar, yet different genetic makeup compared to the rest of Asia. Aside from UGT1A1, variants of SLCO1B1 have also been known to contribute to the severity of neonatal hyperbilirubinemia in Asian populations. Since there has been no report on SLCO1B1 polymorphism in relation with hyperbilirubinemia in Indonesia, this study aims to explore incidence of SLCO1B1*1B polymorphism in Indonesia based on 3 hospitals from different provinces and population, and their association with hyperbilirubinemia severity.

**Methods:**

Our study included 88 neonates with mild and moderate-severe hyperbilirubinemia from 3 NICU in hospitals representing homogenous and heterogenous populations: Biak General Hospital Papua, Cipto Mangunkusumo Hospital (Jakarta), and M Yunus Hospital (Bengkulu). We collected samples between November 2016 and September 2017. DNA was obtained from existing samples of the patients from previous studies and were subjected to Polymerase Chain Reaction – Restriction Fragment Length Polymorphism (PCR-RFLP). We analyzed the *1B variant located in exon 5 of SLCO1B1 with *Taq*I restriction endonuclease. Clinical, demographic, and laboratory data was also collected from medical records and parents’ interviews.

**Results:**

The most dominant variant of SLCO1B1*1B in our population is the homozygous G/G (68.18%), followed by heterozygous A/G (26.14%), and wild type A/A (5.68%). The heterozygous A/G had an Odds Ratio (OR) of 0.73 (95% CI 0.10–5.2) and homozygous G/G with OR of 0.51 (95%CI 0.08–3.27), both were not significant. Genotypic distribution across the different centers were also similar and not significant. The significant risk factors for moderate-severe hyperbilirubinemia were the population the neonate originated from (*p* = < 0.001) and the delivery location (*p* = 0.001), while SLCO1B1*1B was not associated with the different severity of hyperbilirubinemia.

**Conclusions:**

SLCO1B1*1B is not associated with higher bilirubin levels among neonates with hyperbilirubinemia in Indonesia. Further study is needed to find other potentially important genetic polymorphisms in the development of severe hyperbilirubinemia in Indonesia.

## Introduction

Neonatal hyperbilirubinemia (jaundice) occurs in about 60% of all term infants [1]. In general, most of the increase in bilirubin levels is physiological and usually will improve by the end of the first week of life. An increase in total serum bilirubin level above 5 mg/dL can cause jaundice that is visible to observers [2]. This condition can be severe if the infant was born prematurely, where the incidence increases to 80% [2]. In these infants, hyperbilirubinemia can develop into severe neonatal jaundice (SNNJ) that can cause bilirubin-induced neurological damage (BIND) and ultimately lead to irreversible neurodevelopmental impairment or even death [3].

Aside from prematurity, genetic polymorphisms have been reported as an important factor in the development of SNNJ [4–6]. The most commonly-studied gene is the uridine diphosphate glucuronosyltransferase 1A1 (UGT1A1) that is closely associated with Crigler-Najjar’s syndrome type I and II, and Gilberts syndrome. These conditions are frequently found in Caucasian and African populations [7, 8]. Another gene that has been gaining attention, one that is mostly found in Asian populations, is OATP2/SLCO1B1 [9]. SLCO1B1 (solute carrier organic anion transporter family member 1b1) encodes the solute carrier organic anion transporter family member 1B1 and is responsible for the absorption of unconjugated and conjugated bilirubin in the liver. This gene, located in chromosome 12p12, consists of 15 exons (1 coding exon and 1 non-coding exon) and 14 introns, as well as containing 2073 nucleotides in the gene-coding area [9]. Neonates carrying this gene polymorphism are associated with reduced bilirubin clearance which induces hyperbilirubinemia [10]. Rotor Syndrome, an autosomal recessive disease associated with elevated conjugated bilirubin, is an example of a disease frequently associated with mutations in *SLCO1B1* and *SLCO1B3.* The most frequent polymorphisms found in this gene are SLCO1B1*1B (388A > G), SLCO1B1*4 (463C > A), and SLCO1B1*5 (521 T > C), and the most commonly associated with hyperbilirubinemia is the SLCO1B1*1B (388A > G) [9, 11–13].

Based on previous studies, there is an association between *SLCO1B1* with racial variety in regard to the severity of unconjugated neonatal hyperbilirubinemia. In Indian, Japanese, Taiwanese, and Chinese neonates, the incidence of polymorphism is higher in infants with higher bilirubin levels compared to those with lower bilirubin levels [9, 11, 12, 14]. However, in studies of Malaysian and Thai hyperbilirubinemic neonates, this SNP was not frequently found [15, 16]. In Indonesia, polymorphism studies on neonatal hyperbilirubinemia has yet to be conducted extensively. Therefore, this study provides valuable insight into the association of genetic factors especially SLCO1B1 with the severity of neonatal hyperbilirubinemia in Indonesia.

## Materials and methods

### Study population

We conducted a cross-sectional study of patients in neonatal intensive care unit across three hospitals; M. Yunus General Hospital Bengkulu, Biak General Hospital Papua, and Cipto Mangunkusumo Hospital (CMH) Jakarta based on total population sampling. We collected samples and clinical data from November 2016 to September 2017, followed by molecular analysis in 2018. Diagnosis of hyperbilirubinemia was based on clinical jaundice as observed by a neonatologist based on Kramer’s index between day 3–7 since birth before receiving phototherapy, and peak Total Serum Bilirubin (TSB) was then taken prior to initiation of phototherapy. Mild hyperbilirubinemia was considered below 13 mg/dL, and moderate to severe was considered above and equal to 13 mg/dL [17]. Both inborn and outborn, preterm and term infants were included. Patients with hemolytic anemia, cephalhematoma, neonatal sepsis, ABO and rhesus incompatibility, maternal diabetes, and other comorbidities that would affect the total serum bilirubin level were excluded through clinical and laboratory approach. Clinical and sociodemographic variables were obtained through interviews and medical records. Informed consent was obtained from both parents prior to inclusion into the study. This study was approved by the Ethics Committee of Faculty of Medicine, Universitas Indonesia.

### Laboratory investigations

Capillary blood samples were obtained from 88 infants and were used to measure their peak total serum bilirubin using the ADVIA Chemistry Total Bilirubin 2 device (diazo method) [18] taken between day 3 and 7 before phototherapy. A blood sample was also collected from each infant and stored at − 20 °C prior to DNA extraction.

### DNA extraction and manipulation

DNA extraction from blood samples was conducted using the QIAmp Blood DNA Mini Kit (QIAGEN, Germany) according to the manufacturer’s protocol as previously described by Amandito et al. [19]. Following extraction, the DNA concentration was then measured by Nano-spectrophotometer (Thermo-Scientific) for use in polymerase chain reaction - restriction fragment length polymorphism (PCR-RFLP) analysis for determining the SNP in the exon 5 of SLCO1B1.

As much as 2uL of DNA was amplified using KOD FX Neo Kit (Toyobo) with each 25uL mix containing 60 ng extracted DNA, 12.5 uL KOD FX Neo Buffer, 5uL dNTPs (2 mM each of dATP, dCTP, dGTP, dTTP), 0.75uL of each primer (final concentration 8 uM), and 0.7 uL KOD FX Neo DNA polymerase (Novagen, Germany). Amplification was carried out with the following parameters: initial denaturation for 2 min at 95 °C, followed by 35 cycles of 1 min denaturation at 94 °C, annealing for 30 s at 57 °C, extension for 30 s at 68 °C, and final polymerisation for 5 min at 68 °C, and hold at 8 °C for 60 min (Biometra, Germany). All PCR products were visualized by agarose gel electrophoresis in 2% (wt/vol) agarose gels with TBE buffer.

**Fig. 1:**
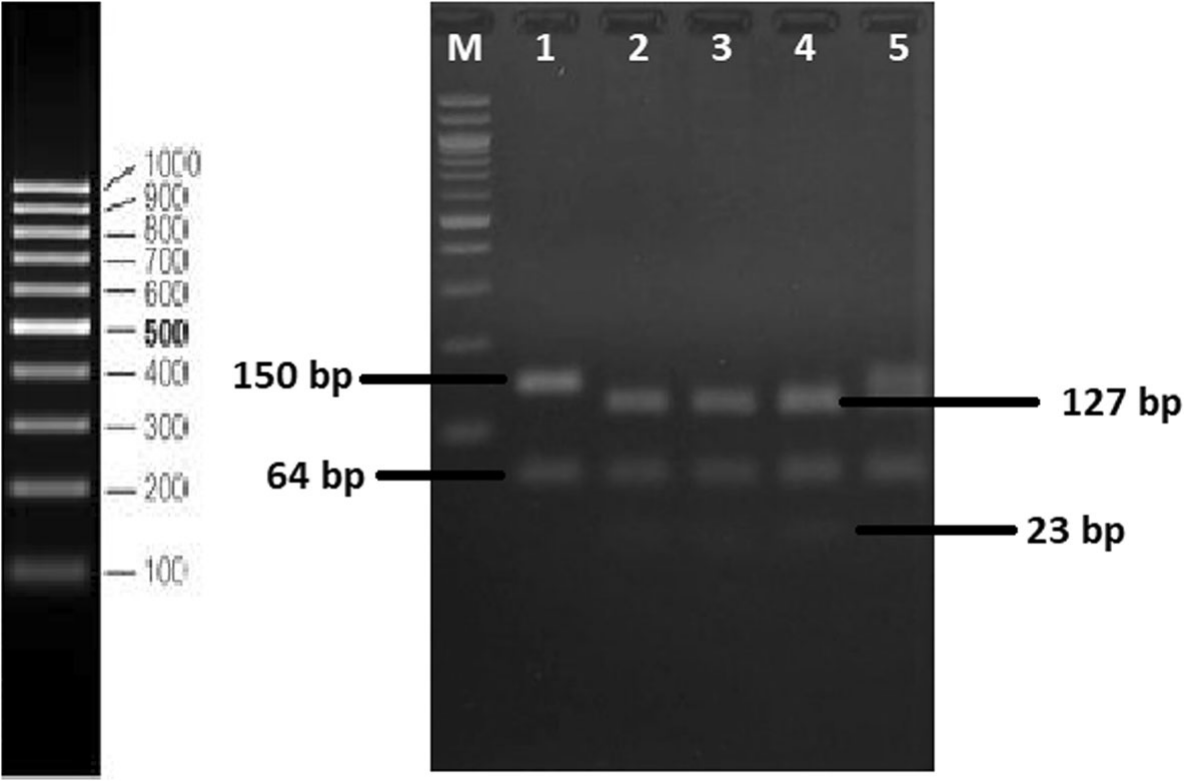
Amplicon from PCR of SLCO1B1 rs 2,306,283 showing a band at approximately 127 bp. Agarose gel electrophoresis after amplicon being digested with *TaqI* restriction endonuclease showed a band at 23 bp in addition to the 127-bp band

To detect the 388A > G SNP (rs2306283), as according to a previous study, we used the primers 388F 5′-ATA ATG GTG CAA ATA AAG GGG-3′ (IDTDNA) for Forward Primer and 388R 5′-ACT ATC TCA GGT GAT GCT CTA-3′ (IDTDNA) for Reverse Primer [15]. For digestion, we used the restriction endonuclease *Taq*I (NEB) and CutSmart buffer (NEB). The digestion procedure consisted of incubation at 65 °C, followed by enzyme inactivation in 80 °C in a Thermal Cycler PCR MJ Mini (Bio-Rad). The sequenced products that were digested were analyzed by electrophoresis on 3% agarose gels with TBE buffer (Fig. 1).

### Statistical analysis

We used SPSS version 24 for data management and statistical analysis. We conducted descriptive analysis for our study prior to inclusion for analytical study. We conducted bivariate analysis between independent variables and dependent variables using parametric and non-parametric methods, as appropriate. Variables with *p* value < 0.25 were included in the multivariate analysis using multiple logistic regression. We used two-sided *p* values in our analysis. Gender, population, exclusive breastfeeding, delivery method, delivery location, sibling requiring phototherapy, and SNPs were categorical data, while gestational age, birth weight, and mother’s age were numerical data.

## Results

### Clinical characteristics

We enrolled 88 hyperbilirubinemic neonates from three different hospitals; Bengkulu (a west coast city on Sumatra Island), Jakarta (capital of Indonesia located on the island of Java), and Biak (a far-eastern remote town on Papua Island), consisting of 47 in the control group (mild hyperbilirubinemia), and 41 in the case group (moderate-severe hyperbilirubinemia). Clinical data of all the patient groups based on the severity of hyperbilirubinemia are shown in Table 1. The mean bilirubin level of all neonates was 13.96 mg/dL with a SD of 6.57, mean gestational age of 33.23 weeks for control and 35.76 weeks for case group, median birth weight of 1580 g for the control group and 2500 g for the case group, and comparable gender between the two groups. Population, delivery location, gestational age, and birth weight were statistically-significant risk factors for moderate-severe hyperbilirubinemia. On the other hand, gender, exclusive breastfeeding, delivery method, sibling requiring phototherapy, and age of the mother were not significant risk factors, and therefore these factors were not included in the multivariate analysis.

**Table 1:** Demographic and clinical data of control vs. case neonates enrolled in the study

Factor in study	Control group	Case group	*P*
Gender (*n* = 88)			
Male	24 (51.1%)	17 (41.5%)	NS^*^
MFemale	23 (48.9%)	24 (58.5%)	
Population (*n* = 88)			
Jakarta	42 (89.4%)	14 (34.1%)	< 0.001^*^
Bengkulu	2 (4.3%)	24 (58.5%)	
Papua	3 (6.4%)	3 (7.3%)	
Exclusive Breastfeeding (*n* = 88)			
Yes	27 (57.4%)	17 (41.5%)	NS^*^
No	20 (42.6%)	24 (58.5%)	
Delivery Method (*n* = 88)			
Vaginal	14 (29.8%)	18 (43.9%)	NS^*^
Caesarean	33 (70.2%)	23 (56.1%)	
Delivery Location (*n* = 88)			
Midwife	2 (4.3%)	12 (29.3%)	0.001^*^
Hospital	45 (95.7%)	29 (70.7%)	
Sibling Requiring Phototherapy (*n* = 88)			
Yes	3 (6.4%)	3 (7.3%)	NS^*^
No	44 (93.6%)	38 (92.7%)	
Gestational age (*n* = 88)			
(Mean + SD, wk)	33.23 + 3.3	35.76 + 3.2	0.001**
Birth weight (*n* = 88)			
(Median (Min-Max), g)	1580 (700–3660)	2500 (940–4100)	< 0.001***
Mother’s age (*n* = 88)			
(Median (Min-Max), y)	29 (18–46)	28 (19–43)	NS***

^*^Chi-square

**Student’s t-test

***Mann-Whitney U-test

For the multiple logistic regression analysis (Table 2), the most significant factor was population with OR of 4.62 (95% CI 1.79–11.97) and *p* value of < 0.001, while delivery location, gestational age, and birth weight were no longer significant.

**Table 2:** Variables in the logistic regression analysis (*n* = 88)

Factor in study	OR (95% CI)	*P*
Population	4.62 (1.79–11.97)	< 0.001
Delivery location	4.26 (0.77–23.5)	NS
Gestational age	1 (0.79–1.31)	NS
Birth weight	1.01 (1–1.02)	NS

### Detection of SLCO1B1*1B polymorphism

Table 3 shows the genotypic frequencies of SLCO1B1*1B variants due to the SNP in exon 5. In the control group, most of the SNPs were homozygote G/G with 34 neonates (72.3%), and wildtype A/A was the least common with only 2 neonates (4.3%). The same is in the case group with 26 neonates (63.4%) homozygote G/G and 3 neonates (7.3%) were wild-type A/A. No statistical significance was achieved when analyzing the two groups based on SLCO1B1 SNP variant. Table 4 shows the analysis between SNPs and bilirubin level which shows no statistical significance.

**Table 3:** Genotypic frequencies in SLCO1B1*1B in control and case groups (*n* = 88)

Factor in study	Control group	Case group	Odds Ratio (95% CI)	*P*
Wildtype A/A	2 (4.3%)	3 (7.3%)	Reference	NS*
Heterozygote A/G	11 (23.4%)	12 (29.3%)	0.727 (0.102–5.2)	
Homozygote G/G	34 (72.3%)	26 (63.4%)	0.51 (0.079–3.27)	

*Fisher’s exact test

**Table 4:** Correlation between bilirubin level and genetic polymorphism (*n* = 88)

SNP	TSB ((Median (Min-Max), mg/dL))	*P*
Wildtype A/A	14.98 (8.3–18.6)	NS*
Heterozygote A/G	13.2 (6.41–29)	
Homozygote G/G	12.39 (5.55–26.7)	

*Kruskal-Wallis

Table 5 highlights the distribution of SLCO1B1*1B between the three centers which represents three different ethnicities and environments, with CMH, Jakarta being the metropolitan capital city with heterogenous ethnicity, M. Yunus, Bengkulu an isolated west coast city with a homogenous ethnicity, and Biak, a remote east town on Papua, also with a homogenous ethnicity. In all three centers, homozygote variant of SLCO1B*1B was the most dominant (75, 57.7, and 50%, respectively), with the heterozygotic variant being the second-most dominant and the wild-type being the least dominant. There was no significant association between the population and the pattern of SLCO1B1*1B polymorphic variance. Table 6 shows the demographic data across the three different hospitals. Between them, only gender (*p* = 0.004) and ethnicity (*p* < 0.001) were significantly different.

**Table 5:** Genotypic distribution of SLCO1B1*1B across three centers (*n* = 88)

Center	Wildtype A/A	Heterozygote A/G	Homozygote G/G	*P*
CMH, Jakarta	2 (3.6%)	12 (21.4%)	42 (75%)	NS*
M. Yunus, Bengkulu	2 (7.7%)	9 (34.6%)	15 (57.7%)	
Biak, Papua	1 (16.7%)	2 (33.3%)	3 (50%)	

*Chi-square

**Table 6:** Demographic data across the three hospital populations

Factor in study	CMH, Jakarta	M. Yunus, Bengkulu	Biak, Papua	*P*
Gender (*n* = 88)				
Male	32 (57%)	5 (19%)	4 (67%)	0.004*
Female	24 (43%)	21 (81%)	2 (33%)	
Exclusive Breastfeeding (*n* = 88)				NS*
Yes	31 (55%)	10 (38%)	3 (50%)	
No	25 (45%)	16 (62%)	3 (50%)	
Delivery Location (*n* = 88)				NS*
Midwife	52 (93%)	17 (65%)	5 (83%)	
Hospital	4 (7%)	9 (35%)	1 (17%)	
Ethnicity (*n* = 88)				< 0.001*
Javanese	19 (34%)	0 (0%)	0 (0%)	
Betawi	15 (27%)	0 (0%)	0 (0%)	
Sundanese	13 (24%)	0 (0%)	0 (0%)	
Minangkabau	6 (10%)	0 (0%)	0 (0%)	
Indo-Chinese	3 (5%)	0 (0%)	0 (0%)	
Bengkulu	0 (0%)	26 (100%)	0 (0%)	
Papuan	0 (0%)	0 (0%)	6 (100%)	
Gestational age (*n* = 88)				NS**
(Mean + SD, wk)	33.66 + 3.26	35.42 + 3.35	37 + 4.65	
Birth weight (*n* = 88)				NS**
(Median (Min-Max), g)	1887 (910–4100)	2500 (1200–3700)	2890 (700–3500)	
Mother’s age (*n* = 88)				NS**
(Median (Min-Max), y)	31.5 (18–43)	26 (19–38)	28 (21–46)	

^*^Chi-square

**Kruskal-Wallis

We conducted additional analysis from both groups and within each group to find any association between SLCO1B1*1B and bilirubin level and found there was no significant association.

## Discussion

Physiological conditions such as the short neonatal red blood cell’s lifespan, absorption of bilirubin by the liver and a non-optimal conjugation process of bilirubin can cause hyperbilirubinemia [1, 2]. Premature birth carries a higher risk of severe hyperbilirubinemia because of the immaturity of the red blood cells and an impaired hepatic cell function responsible for neonatal the bilirubin metabolism in neonates [2, 20]. The cause for this condition also varies between populations due to suspected contributions of genetic factors towards the pathogenesis of hyperbilirubinemia.

SLCO1B1 is a sinusoidal membrane protein expressed at the basolateral membrane of hepatocytes and is putatively involved in facilitating the hepatic uptake of unconjugated bilirubin [9, 13]. The exon 5 mutation of 388G > A is a nonsynonymous SNP with a missense mutation that causes a single amino acid substitution (asparagine to aspartic acid) encoded by codons 130 of SLCO1B1. This change disrupts the function of the SLCO1B1 transporter in transporting bilirubin from blood to the liver, leading to bilirubin concentration remaining high in the blood, which, in turn, causes hyperbilirubinemia [12].

Aside from the well-established risk factors of low gestational age and low birth weight, we found a significant association between the severity of hyperbilirubinemia and difference of populations across Jakarta, Bengkulu, and Papua, as well as with delivery location.

SLCO1B1*1B in both the heterozygous and homozygous variants was predominantly found (94.32%) in the neonates that we studied. Compared with the study of UGT1A1*60 and UGT1A1*6 from our previous study, SCLO1B1*1B was similar to *60 which showed a high incidence albeit with no significant association with the higher bilirubin level (severity) in neonatal hyperbilirubinemia [19]. Other studies on *SCLO1B1* in different countries also showed similar findings to our study concerning the incidence of polymorphisms. In a study in India, there were 77.7% hyperbilirubinemic neonates having the variant *1B in hyperbilirubinemic neonates, and 87.7% in Malaysia. All these studies demonstrate that more than half of the studied population has the *1B variant [14, 16, 21]. Both Asian countries share a lot in common in terms of genetic makeup with Indonesia.

Regarding the association between SLCO1B1*1B and higher bilirubin level in infants, there are still inconclusive evidence from different studies of different populations. In a study by Huang et al. in 2004, they reported the *1B SNP to be more frequent among hyperbilirubinemic infants with an OR of 2.01. In the study by Liu et al., the Chinese variant of *1B was also found to be associated with hyperbilirubinemia in the Guangdong population, but not in the Yunnan population [9]. Whereas other studies in Fujian and Taiwan also failed to prove the association of *1B with neonatal hyperbilirubinemia, one study in Taiwan showed that Taiwanese neonates with the minor allele of *1B were at high risk to develop severe hyperbilirubinemia [22–24].

Some ethnicities are shown to be more at risk for severe hyperbilirubinemia than others such as African-American and Asian [25]. This is most likely caused by the different prevalence of genetic polymorphisms responsible for metabolism or transport pathways [26]. Variations of specific genes can also differ between the individuals in a smaller population group. An example of this is the intra-ethnic differences of *UGT1A1* polymorphism in the Chinese populations of Han, She, and Dong origin [27]. Global analysis of *SLCO1B1* also suggests that *SLCO1B1* diversity is greater within populations than between populations [28]. Conversely, we did not find any intra-ethnic differences in our three populations for *SLCO1B1* polymorphism.

From the comparison of genotypic distribution and clinical factor among the three different centers comprising population of different sub-ethnic origins, we observed a significant association with the severity of hyperbilirubinemia independent from the history of exclusive breastfeeding and other established clinical risk factors. This suggests the presence of a potentially undiscovered important risk factor for the development severe hyperbilirubinemia in the Indonesian population. Further research effort in Indonesia is warranted to investigate other potential genetic factors in different pathways leading to hyperbilirubinemia, including BLVRA and HMOX1 [29–32].

Our study is limited by the fact that we used a bank of DNA from a larger project, in which some subjects’ DNA samples were not sufficient for genetic analysis. Therefore, we were unable to study the whole population that we originally collected from the three centers. We also did not use consistently homogenous populations in the comparisons, and this could have obscured certain ethnicities where a different polymorphism variant could be dominant instead of *1B. The method of SNP analysis using PCR-RFLP is also less efficient and effective compared to other techniques such as DNA sequencing. However, we believe it is still sufficient in order to obtain national preliminary data that would have been otherwise severely lacking.

## Conclusion

SLCO1B1*1B was found to be a common occurrence in Indonesian hyperbilirubinemic neonates. There was no statistically significant differences between occurrence of the SLCO1B1*1B variant and severity of neonatal hyperbilirubinemia. There was, however, a significant difference in the severity depending on the center where the neonate was enrolled. Future studies are required that includes a more diverse population to represent the entirety of the ethnically-diverse Indonesian population and exploring additional genetic risk factors is highly recommended.

## Data Availability

The datasets generated and/or analysed during the current study are not publicly available due to regulation by the hospital but are available from the corresponding author on reasonable request.

## Abbreviations

CI: Confidence Interval
CMH: Cipto Mangunkusumo Hospital
PCR–RFLP: polymerase chain reaction–restriction fragment length polymorphism
SD: Standard deviation
SLCO1B1: Solute Carrier Organic Anion Transporter Family Member 1b1
SPSS: Statistical package for the social sciences
TSB: Total serum bilirubin
UGT1A1: UDP-glucoronosyltransferase 1A1
